# Incorporating DNA Sequencing into Current Prenatal Screening Practice for Down's Syndrome

**DOI:** 10.1371/journal.pone.0058732

**Published:** 2013-03-20

**Authors:** Nicholas J. Wald, Jonathan P. Bestwick

**Affiliations:** Wolfson Institute of Preventive Medicine, Barts and the London School of Medicine and Dentistry, London, United Kingdom; VU University Medical Center, The Netherlands

## Abstract

**Background:**

Prenatal screening for Down's syndrome is performed using biochemical and ultrasound markers measured in early pregnancy such as the Integrated test using first and second trimester markers. Recently, DNA sequencing methods have been introduced on free DNA in maternal plasma, yielding a high screening performance. These methods are expensive and there is a test failure rate. We determined the screening performance of merging the Integrated test with the newer DNA techniques in a protocol that substantially reduces the cost compared with universal DNA testing and still achieves high screening performance with no test failures.

**Methods:**

Published data were used to model screening performance of a protocol in which all women receive the first stage of the Integrated test at about 11 weeks of pregnancy. On the basis of this higher risk women have reflex DNA testing and lower risk women as well as those with a failed DNA test complete the Integrated test at about 15 weeks.

**Results:**

The overall detection rate was 95% with a 0.1% false-positive rate if 20% of women were selected to receive DNA testing. If all women had DNA testing the detection rate would be 3 to 4 percentage points higher with a false-positive rate 30 times greater if women with failed tests were treated as positive and offered a diagnostic amniocentesis, or 3 times greater if they had a second trimester screening test (Quadruple test) and treated as positive only if this were positive. The cost per women screened would be about one-fifth, compared with universal DNA testing, if the DNA test were 20 times the cost of the Integrated test.

**Conclusion:**

The proposed screening protocol achieves a high screening performance without programme test failures and at a substantially lower cost than offering all women DNA testing.

## Introduction

The testing of cell-free DNA circulating in maternal plasma offers an effective means of screening for Down's syndrome with detection rates (proportion of affected pregnancies with positive results) of 98% or more and false-positive rates (proportion of unaffected pregnancies with positive results) of about 0.2% or less [1]–[7]. At present such DNA testing tends to be expensive (cited as being charged $795 to $2762 in the United States [6]) and requires specialist expertise available in only a few laboratories. In about 2–13% of pregnancies a result cannot be obtained for various reasons including insufficient fetal DNA in the maternal plasma. These can be referred to as test failures and tend to be ignored in describing the efficacy of the test. One study [1] reported 11.5% (compromised sample, haemolyzed sample, inadequate volume, failed quality control). Another reported a test failure rate of 3.4% (0.88% assay failure and 2.50% of samples inadequate [2]). A third study reported that 16 out of 532 pregnancies (3%) yielded insufficient fetal DNA and could not be tested and 4 out of 96 Down's syndrome pregnancies (4.2%; 3 mosaics) yielded an unidentifiable result as well as 24 out of 426 unaffected pregnancies (5.6%) suggesting a test failure rate of 7% [3]. A fourth study reported that in one group 5% did not meet “QC criteria”, but none in a second group of the same size [4]. In a fifth study based on women undergoing routine screening, results were not obtained on 4.9% of samples [5]. In a sixth study, based on a mixture of women with positive conventional screening tests for Down's syndrome and women undergoing routine screening, 1.6% failed the quality control criteria [5]. In a seventh study 21 out of 166 samples did not pass the DNA quality test (13%) [7].

We here propose a screening protocol arising from, and improving on, an idea previously reported [8], that merges existing methods based on fetal ultrasound measurements and immunoassays with the newer DNA techniques in a way that would substantially reduce the cost compared with the cost of universal DNA testing and still achieve a high screening performance, that is, a high detection rate for a low false-positive rate.

**Figure 1:**
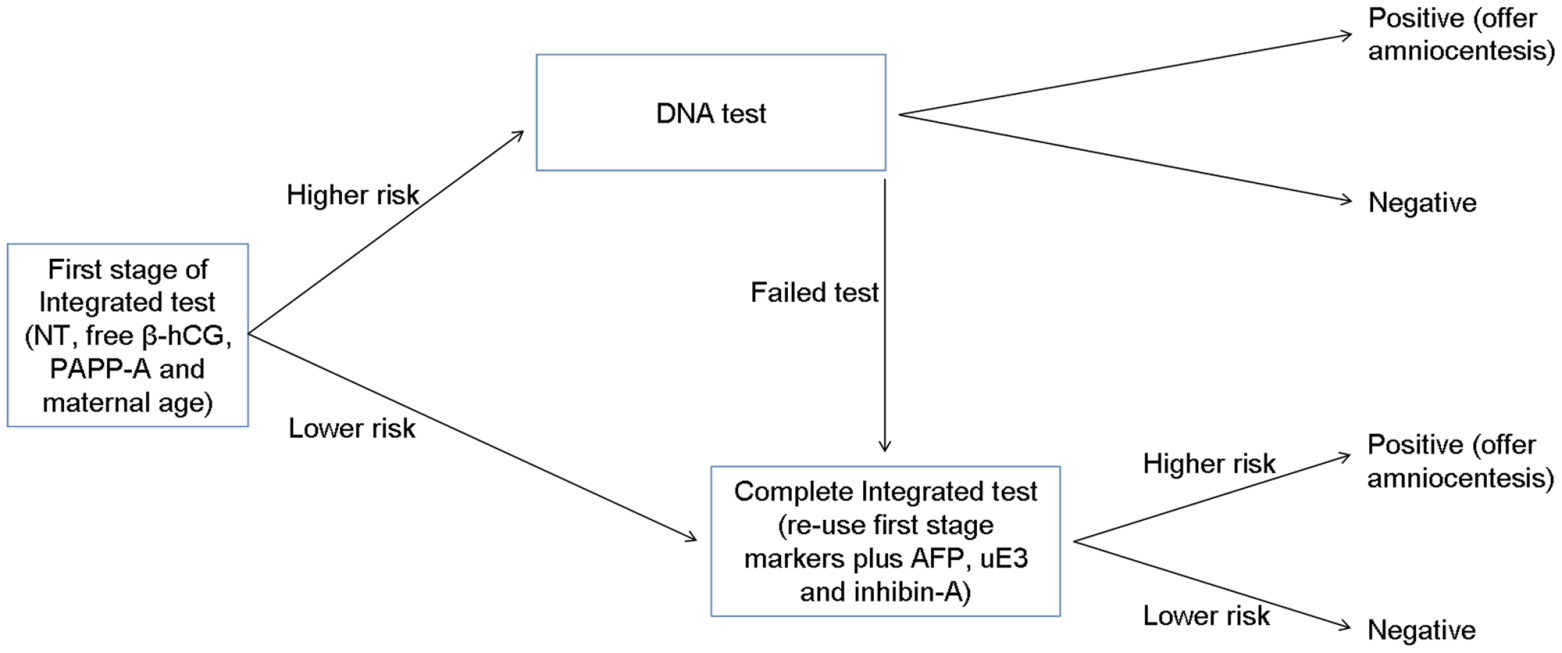
Protocol for reflex DNA testing in conjunction with Integrated test screening.

The proposed protocol, which is outlined in Figure 1, uses the first trimester stage of an Integrated test (late first trimester measurement of the ultrasound marker nuchal translucency [NT] and the serum markers free β-human chorionic gonadotropin [hCG] and pregnancy associated plasma protein-A [PAPP-A] with maternal age; the Combined test) to determine which women would receive an automatic DNA sequencing test on the sample already collected. The automatic application of a second test in this way can be referred to as “reflex” DNA testing, ie. one triggered by the result of the first test. All women who do not receive a DNA test result proceed to have the second part of the Integrated test which includes the re-use of maternal age and the NT, free β-hCG and PAPP-A measurements together with early second trimester measurement of the serum markers alphafetoprotein [AFP], unconjugated estriol [uE_3_] and inhibin-A. Typically the Integrated test uses an hCG (total or the free β) measurement in the second trimester, but in the proposed protocol it is measured in the first trimester instead.

The proposal provides a practical solution to the problem that while DNA testing is an effective screening method it is expensive and can be done only at a small number of laboratories. We offer a solution to this by using the existing screening tests based on serum markers and an ultrasound marker used at a very low false-positive rate to identify a higher risk group, such that only the higher risk women need to have the DNA testing. Importantly, this entails little loss in screening performance.

## Methods

Screening performance of the protocol was estimated as follows. Multivariate Gaussian distribution parameters (means, standard deviations and correlation coefficients) of the screening markers from a large cohort study (the Serum Urine and Ultrasound Screening Study [SURUSS]) [9], revised to incorporate subsequent improvements [10], were used to simulate 500,000 Down's syndrome pregnancies and 500,000 unaffected pregnancies, each with a set of marker values (first trimester marker values measured at 11 completed weeks' gestation). Each simulated pregnancy was assigned a maternal age based on the distribution of maternities in England and Wales from 2006 to 2008 inclusive (the latest available at the time the study was performed) [11] and the maternal age-specific odds of an affected livebirth [12]–[14].

For each simulated pregnancy, a risk of being affected with Down's syndrome based on the first stage of the Integrated test (NT, free β-hCG and PAPP-A) was calculated by multiplying the maternal age-specific odds of having an affected live birth (adjusted to early second trimester by multiplying by 1/0.77 to allow for the general fetal loss in Down's syndrome pregnancies from this time in pregnancy until term [14]) by the likelihood ratio for being affected (for the simulated set of marker values) which was calculated from the multivariate Gaussian distributions of NT, free β-hCG and PAPP-A levels in affected and unaffected pregnancies. The risk cut-offs that yielded initial false-positive rates of 10%, 20%, 40%, 60%, 80% and 90% were determined. A DNA result for those with a risk greater than or equal to the calculated risk cut-off levels was generated. The test failure rate was taken as 3% (towards the lower range of estimates because in some instances a repeat sample may be obtained and provide a test result and these are not considered as test failures in our analyses). 97% were assigned a successfully completed DNA test and 3% were not. Of those in which DNA testing was assumed to have been successfully completed, screening performance was taken from Palomaki et al. [2] as being typically within the range of estimates, so 98.6% of the simulated Down's syndrome pregnancies and 0.2% of the simulated unaffected pregnancies were randomly classified as being screen positive based on DNA sequencing. For those initially classified low risk, and for those in whom DNA testing failed, an Integrated test risk of being affected with Down's syndrome was calculated re-using the first trimester markers together with the second trimester markers. Those with an Integrated test risk greater than 1 in 50 were classified as being screen positive. Overall detection and false-positive rates were estimated, and compared with estimates based on all women having a DNA test. The estimated screening performance was compared with universal DNA testing based on (i) offering women with failed tests a diagnostic test, treating them as screen positive, so yielding a 3.0% false-positive rate, which with the 0.2% test false-positive rate sums to 3.2% or (ii) offering women a second trimester Quadruple test (AFP, uE_3_, hCG and inhibin-A; 85% detection rate for a 5% false-positive rate) [10]. A similar analysis was done based on using the Combined test (NT, free β-hCG and PAPP-A) only.

We estimated the cost of the screening protocol per woman as a multiple of the cost of an Integrated test. If *C_IT_* is the cost of an Integrated test, *C_DNA_* the cost of a DNA test, *a* the proportion of the Integrated test cost incurred in the first trimester, *b* the proportion of the Integrated test cost incurred in the second trimester and *P* the proportion of women who have DNA testing (i.e. the positive rate of the initial stage of the Integrated test), the cost per woman screened is:-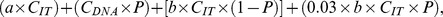
i.e. the cost of the first stage of an Integrated test, plus the cost of a DNA test in women who have a DNA test plus the cost of the second stage of the Integrated test in women who do not have a DNA test plus the cost of the second stage of the Integrated test in women who have a DNA test, but the test failed.

Because *a*+*b* = 1, *b* = 1-*a*, and after dividing throughout by *C_IT_* and rearranging, the cost per woman screened as a multiple of the cost of the Integrated test is given by the following equation
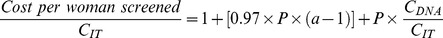



So if *a* is 75%, *P* is 10% and the DNA test is 20 times more expensive than the Integrated test, the cost per woman screened as a multiple of the Integrated test cost is 1+[0.97×0.1×(0.75−1)]+20×0.1 = 3.0.

A similar analysis was performed using the protocol applied to the Combined test.

The cost per woman screened as a multiple of the Combined test is 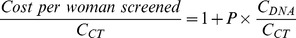



## Results

**Figure 2:**
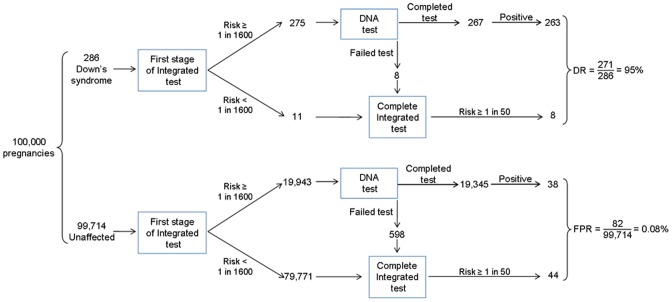
Protocol for reflex DNA testing in conjunction with Integrated test screening in which 20% of women at highest risk using NT, PAPP-A, and free β-hCG (first part of the Integrated test) receive a DNA sequencing test.

Figure 2 is a flow diagram showing an example of the effect of DNA sequencing in conjunction with the Integrated test in 100,000 pregnancies (including 286 with Down's syndrome based on the early second trimester prevalence [11]–[15]). The first trimester risk cut-off to achieve an initial false-positive rate of 20% based on NT, free β-hCG, and PAPP-A was 1 in 1,600. Women with a risk estimate greater than or equal to this risk cut-off have a reflex DNA test. A test result is not reported at this stage. The remaining women continue to have the second part of the Integrated test. The figure shows 275 (96%) affected and 19,943 (20%) unaffected pregnancies have a reflex DNA test. For 3% of these women the DNA test fails (8 affected, 598 unaffected). These women have second trimester markers measured and an Integrated test risk is reported. Among women who have a completed DNA test, 263 affected and 38 unaffected pregnancies have a positive result. Among the women whose DNA test failed and the women who were not selected for DNA testing after the first trimester stage of the Integrated test, 8 affected and 44 unaffected pregnancies are positive based on the completed Integrated test (risk ≥1 in 50). The overall screening performance is an estimated detection rate of 95% (271/286) with a false-positive rate of 0.1% (82/99,714).

Table 1 shows the screening performance of this approach according to the percentage of women selected for a reflex DNA test based on the first stage of the Integrated test. As the percentage increases from 10% to 90% the detection rate increases from about 92% to 98% and the false-positive rate initially decreases from 0.11% (10% have a DNA test) to 0.08% (20% have a DNA test) then increases to 0.20% (90% have a DNA test). Table 1 also shows the screening performance of routine DNA testing without an Integrated test if (i) women with a DNA test failure have a diagnostic amniocentesis or (ii) women with a DNA test failure have a second trimester screening Quadruple test, the two practical options available. In the first case DNA test failures are regarded as screen-positive and the detection rate is 98.6% with a false-positive rate of 3.2%. In the second case, in which women with a failed test have a Quadruple test, the detection rate is 98.0% with a false-positive rate of 0.3%.

**Table 1:** Screening performance of reflex DNA tests with the Integrated test according to percentage of women having reflex DNA test (Integrated test risk cut-off 1 in 50).

Women selected for reflex DNA test after first stage of Integrated test	Risk cut-off for first stage of Integrated test	Overall screening performance	
		Detection rate (%)	False-positive rate (%)
10%	1 in 630	92.4	0.11
20%	1 in 1600	94.8	0.08
40%	1 in 4900	96.9	0.10
60%	1 in 12000	97.7	0.14
80%	1 in 27000	98.1	0.18
90%	1 in 47000	98.2	0.20
All women have a DNA test (no Integrated test):-			
test failures classified as positive		98.6	3.19
test failures have a Quadruple test, risk cut-off 1 in 100		98.0	0.29

Table 2 shows, in a similar way to Table 1, the cost per woman screened expressed as a multiple of the cost of an Integrated test. As the percentage of women having reflex DNA testing increases from 10% to 90% the cost increases from double to ten-fold if the cost of the DNA test is ten times the cost of the Integrated test. Table 2 also shows how the cost per woman screened changes as the cost of the DNA test decreases. For example if the cost of the DNA test were 20 times the cost of the Integrated test, and 20% of women classified as higher risk at the first stage of the Integrated test, the cost per woman screened would be 5.0 times greater than the cost of the Integrated test. If the cost of the DNA test were 10 times the cost of the Integrated test, the cost per woman screened would be 3.0 times greater. The estimates in Table 2 are based on 75% of the cost of the Integrated test being incurred at the first trimester stage but are robust to different proportions. For example with the cost being split equally between the first and second trimesters the cost per woman screened would be 4.9 instead of 5.0 and 2.9 instead of 3.0 times greater in the examples above.

**Table 2:** Illustration of the cost per woman screened according to the cost of the DNA test, expressed as a multiple of the cost of an Integrated test, and the proportion of women who have a DNA test (i.e. positive based on a the first trimester stage of the Integrated test, 75% of the cost of the Integrated test incurred in the first trimester).

Women selected for reflex DNA test after first stage of Integrated test	DNA test cost as a multiple of Integrated test cost				
	2.5	5	10	20	40
10%	1.2	1.5	2.0	3.0	5.0
20%	1.5	2.0	3.0	5.0	9.0
40%	1.9	2.9	4.9	8.9	17
60%	2.4	3.9	6.9	13	25
80%	2.8	4.8	8.8	17	33
90%	3.0	5.3	9.8	19	37

Figures S1 and S2 illustrate the effect of using the reflex DNA testing approach with the Combined test in the same way that figures 1 and 2 do with the Integrated test, and Tables S1 and S2 present the corresponding estimates in the same way as tables 1 and 2. The screening performance is similar to that with the Integrated test.

## Discussion

The reflex DNA testing protocol proposed here, in which the first part of an Integrated test is used to determine who should receive a DNA test, has a number of advantages. First, it has a high screening performance; while the detection rate may be about five percentage points lower than routine DNA testing, the false-positive rate is about 30 times lower if DNA test failures are considered positive and these women have a diagnostic amniocentesis, or about 3 times lower if these women have a second trimester Quadruple test. Second, all women receive a screening result; there are no failed tests and there is no need to tell some women that they had a failed DNA test and needlessly cause them anxiety. Third, the cost of DNA sequencing for the programme is reduced by 80 or 90%, depending on which of the options is adopted. The cost of continuing the Integrated test would remain, which in a public service context is available at the Wolfson Institute of Preventive Medicine, London, for £35 (about $50), but at present this is substantially less than all women having a DNA test. Fourth, the very low false positive rate means that only about 3 per 1000 women screened would need a diagnostic amniocentesis and in about 2 in 3 a Down's syndrome pregnancy would be diagnosed. Fifth, such a protocol allows for the screening of other pregnancy complications such as pre-eclampsia, identified using immunoassays or heart defects using ultrasound markers.

In our analysis we used the results of Palomaki and colleagues (DNA test detection rate of 98.6% and false-positive rate of 0.2% [1]) and a DNA test failure rate of 3%. A sensitivity analysis showed that our estimated overall detection rate of 94.8% and overall false-positive rate of 0.08% is robust to reported variations in the DNA test detection rates, false-positive rates, and test failure rates. For DNA detection rates between 97.5% and 99.5%, and false-positive rates between 0.1% and 0.3%, and DNA test failure rates between 1% and 5%, the overall detection rates were between 93.6% and 95.8% and the overall false-positive rate between 0.05% and 0.12%.

In a typical Integrated test free β-hCG is not measured in the first trimester, but either total or free β-hCG is measured in the second trimester. In the proposed protocol, the overall screening performance is marginally better by measuring hCG earlier; if total hCG were measured in the second trimester and only an NT and PAPP-A measured in the first trimester, the overall detection rate would be 93.8% (instead of 94.8%) and the overall false-positive rate 0.12% (instead of 0.08%) if 20% of women were selected for a reflex DNA test. There is no advantage in measuring hCG in both the first and second trimesters. The measurement of additional markers such as serum placental growth factor, or ultrasound ductus venosus blood flow or nasal bone would improve the screening performance of the proposed protocol, but these are not routinely used, and therefore are not considered here.

Most of the published studies on DNA sequencing as a screening test for Down's syndrome were performed on women who were, for one reason or another, at higher than average risk, but this is not a source of bias as the detection rate and false-positive rate of screening tests using markers that are the consequence of the disorder are independent of the prevalence of the disorder.

As experience is gained with the screening protocol proposed in this paper, and if the cost of DNA testing falls, with a lower rate of failed tests, DNA testing could be offered to a larger proportion of pregnancies. At the same time the need for immunoassays and ultrasound measurements currently used to screen for Down's syndrome can be reviewed to assess their value in screening for other disorders such as pre-eclampsia.

Table 2 provides an indication of the costs of the proposed protocol with varying proportions having a DNA test. Expressing cost in multiples of the cost of the Integrated test makes the table generally applicable, so that, if a 4 to 5 fold greater cost per woman screened were acceptable, a 20% DNA testing percentage could be done if the DNA test were 20 times more costly than the Integrated test, or 40% if 10 times more costly. As can be seen from figure 2, the marginal cost of detecting the extra 4% (from 95% to 99%) of Down's syndrome pregnancies that would be detected if all women had DNA testing rather than adopting the proposed protocol would be extremely high, requiring about 80,000 extra DNA tests (100,000 – 20,000) for each extra Down's syndrome pregnancy detected (if 20% have reflex DNA testing). This would remain the case unless the cost of a DNA test were substantially reduced. Collecting plasma samples for DNA testing that may not be used is, of course, an expense, but a small one compared with routine DNA testing.

Reflex DNA testing with the Combined test rather than the Integrated test yields a similar screening performance (see Table S1) with a similar overall cost (see Table S2). The principal disadvantage in using reflex DNA testing with the first trimester Combined test instead of the first stage of the Integrated test is that it would generate a burden of anxiety in a significant number of women. This is because a negative Combined test result would be informed immediately if testing were done at the time of the ultrasound NT measurement or within a day or two if done in a central laboratory. However, because many would regard it to be wrong to artificially delay the reporting of a negative screening result, a “positive” would trigger a DNA test that would take 1–2 weeks, so if women did not receive a prompt result they would realize that they were in a higher risk category. If 20% were selected for a DNA test, as shown in Figure S2, this would apply to 20% of women in the population. If, however, as we suggest DNA testing were conducted together with the Integrated test the corresponding proportion would be substantially less than 1%.

A feature of the proposed screening protocol is that it applies equitably to all pregnant women with the cost averaged over all women screened, regardless of who has a DNA test. This is a practical approach in the context of a screening programme, which is the appropriate way of delivering the service to a population.

Our proposed protocol could usefully be linked to interpretive software that would automatically modify the proportion of women selected for reflex DNA testing and provide the appropriate risk cut-off to achieve this. Also, all women who have a DNA test could receive an estimate of the risk of having a Down's syndrome pregnancy, based on a combination of the DNA test result and the first stage of the Integrated test, so the results would be quantitative and not simply be based on a qualitative DNA result. In deriving this risk, the DNA result could be adjusted for relevant factors such as the fetal DNA fraction and maternal weight.

In summary, the proposed protocol combines current screening methods with the newer DNA sequencing methods to provide a cost effective strategy for all pregnant women with a very high level of efficacy and safety.

## Supporting Information

Figure S1 **Protocol for reflex DNA testing in conjunction with Combined test screening.**
(TIF)

Figure S2 **Protocol for reflex DNA testing in conjunction with Combined test screening in which 20% of women at highest risk using the Combined test receive a DNA sequencing test.**
(TIF)

Table S1 **Screening performance of reflex DNA tests with the Combined test according to percentage of women having reflex DNA test.** (Combined test risk cut-off 1 in 50 for DNA test failures).
(DOCX)

Table S2 **Illustration of the cost per woman screened according to the cost of the DNA test, expressed as a multiple of the cost of a Combined test, and the proportion of women who have a DNA test (i.e. positive based on the Combined test).**
(DOCX)
