# Supplementary figures and images for: Incorporating DNA Sequencing into Current Prenatal Screening Practice for Down's Syndrome
Source: PLoS One. 2013 Mar 20;8(3):e58732. doi: 10.1371/journal.pone.0058732 (PMC3604109; doi:10.1371/journal.pone.0058732)

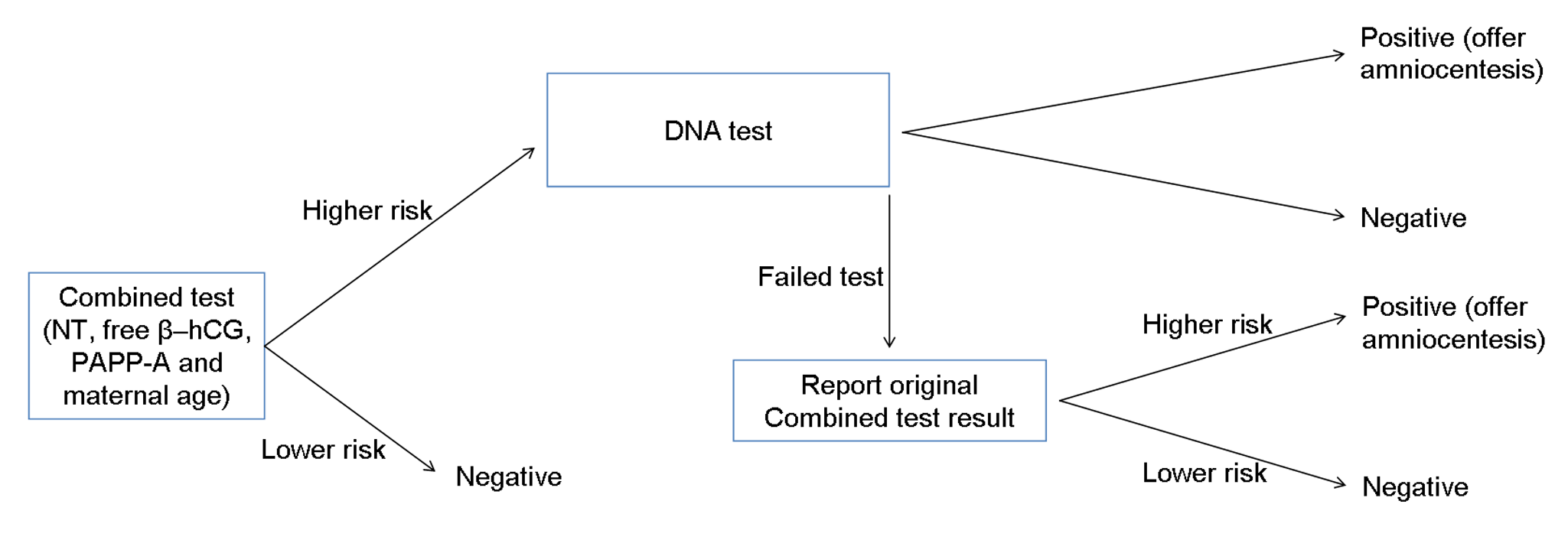

Supplement: Figure S1 — Protocol for reflex DNA testing in conjunction with Combined test screening. (TIF) [file pone.0058732.s001.tif]

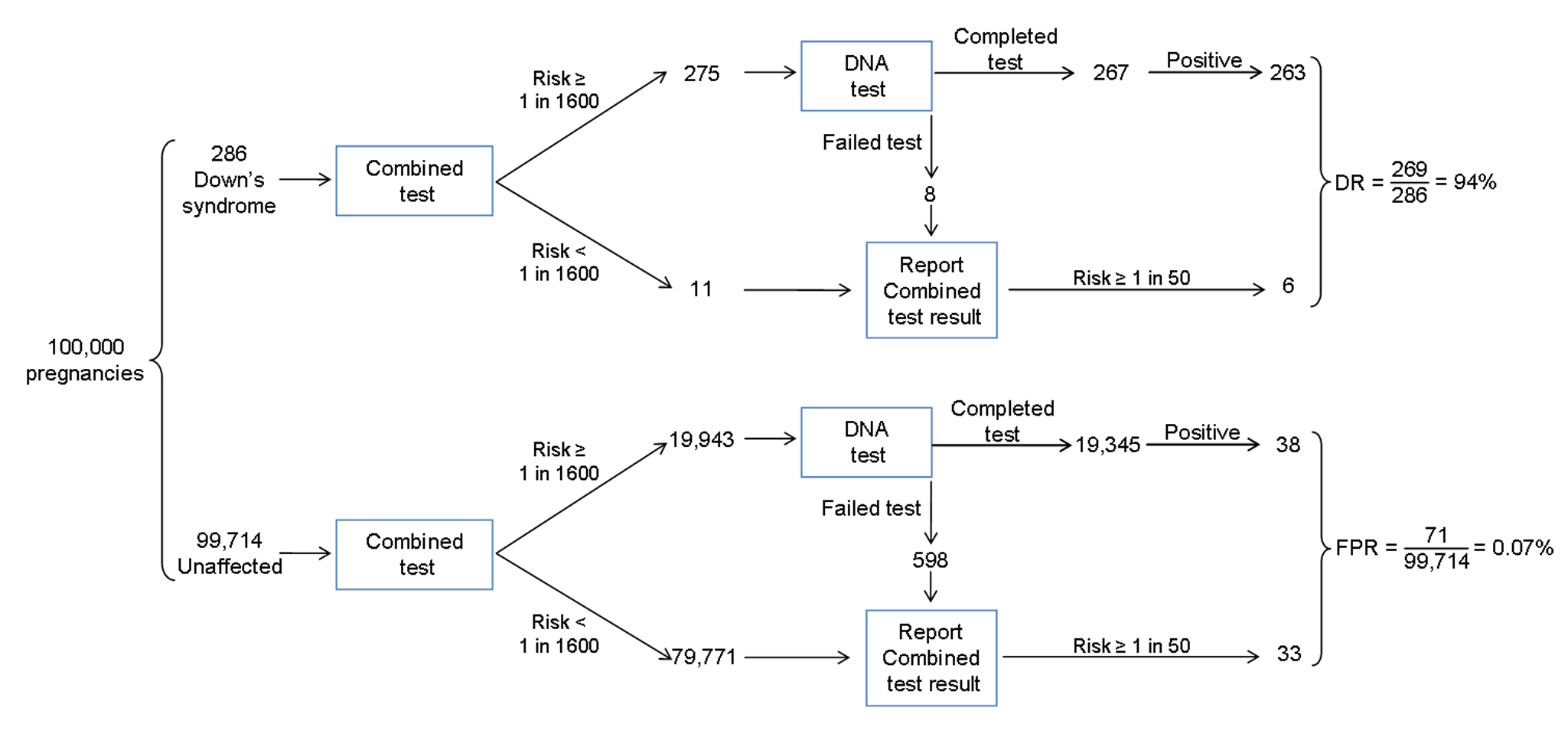

Supplement: Figure S2 — Protocol for reflex DNA testing in conjunction with Combined test screening in which 20% of women at highest risk using the Combined test receive a DNA sequencing test. (TIF) [file pone.0058732.s002.tif]
