# Supplementary material for: Incorporating DNA Sequencing into Current Prenatal Screening Practice for Down's Syndrome
Source: PLoS One. 2013 Mar 20;8(3):e58732. doi: 10.1371/journal.pone.0058732 (PMC3604109; doi:10.1371/journal.pone.0058732)
Supplement: Table S1 — Screening performance of reflex DNA tests with the Combined test according to percentage of women having reflex DNA test. (Combined test risk cut-off 1 in 50 for DNA test failures). (DOCX) [file pone.0058732.s003.docx]

Table S1: Screening performance of reflex DNA tests with the Combined test according to percentage of women having reflex DNA test. (Combined test risk cut-off 1 in 50 for DNA test failures).

| Women selected for reflex DNA test after Combined test | Risk cut-off for Combined test | Overall screening performance | |
| --- | --- | --- | --- |
|  |  | Detection rate (%) | False-positive rate (%) |
| 10% | 1 in 630 | 90.7 | 0.05 |
| 20% | 1 in 1600 | 94.1 | 0.07 |
| 40% | 1 in 4900 | 96.5 | 0.11 |
| 60% | 1 in 12000 | 97.4 | 0.15 |
| 80% | 1 in 27000 | 97.8 | 0.19 |
| 90% | 1 in 47000 | 97.9 | 0.20 |
| All women have a DNA test (no Combined test):- | | | |
| test failures classified as positive | | 98.6 | 3.19 |
| test failures have a Quadruple test, risk cut-off 1 in 100 | | 98.0 | 0.29 |
