# Supplementary material for: Incorporating DNA Sequencing into Current Prenatal Screening Practice for Down's Syndrome
Source: PLoS One. 2013 Mar 20;8(3):e58732. doi: 10.1371/journal.pone.0058732 (PMC3604109; doi:10.1371/journal.pone.0058732)
Supplement: Table S2 — Illustration of the cost per woman screened according to the cost of the DNA test, expressed as a multiple of the cost of a Combined test, and the proportion of women who have a DNA test (i.e. positive based on the Combined test). (DOCX) [file pone.0058732.s004.docx]

Table S2: Illustration of the cost per woman screened according to the cost of the DNA test, expressed as a multiple of the cost of a Combined test, and the proportion of women who have a DNA test (i.e. positive based on the Combined test)

| Women selected for reflex DNA test after first stage of Combined test | DNA test cost as a multiple of Combined test cost | | | | |
| --- | --- | --- | --- | --- | --- |
|  | 2.5 | 5 | 10 | 20 | 40 |
| 10% | 1.3 | 1.5 | 2.0 | 3.0 | 5.0 |
| 20% | 1.5 | 2.0 | 3.0 | 5.0 | 9.0 |
| 40% | 2.0 | 3.0 | 5.0 | 9.0 | 17 |
| 60% | 2.5 | 4.0 | 7.0 | 13 | 25 |
| 80% | 3.0 | 5.0 | 9.0 | 17 | 33 |
| 90% | 3.3 | 5.5 | 10 | 19 | 37 |
